# Prevalence of Depression and Anxiety Disorders Among Cancer Patients: An Insight From a Single Institute

**DOI:** 10.7759/cureus.42831

**Published:** 2023-08-01

**Authors:** Remya Radhakrishnan, Hemalatha Selvaraj, Kumarappan Chidambaram, Arshav KV, Adona James, Sivakumar Thangavel

**Affiliations:** 1 Pharmacy Practice, Nandha College of Pharmacy, Erode, IND; 2 Pharmacology & Toxicology, King Khalid University, Abha, SAU

**Keywords:** cancer, anxiety, anxiety disorders, depression, oncology, psychiatry morbidity, forecasters, breast cancer, hads

## Abstract

Objective

This cross-sectional, observational study aimed at finding the prevalence of anxiety and depression in cancer patients and the correlation of anxiety and depression with various factors, such as age, sex, marital status, educational status, occupation, financial support, duration, type of care, sort of carcinoma, and stages of malignancy, among cancer patients attending the G. Kuppuswamy Naidu Memorial Hospital, Coimbatore, Tamil Nadu, India from July 2022 to December 2022, using the Hospital Anxiety and Depression Scale (HADS).

Methods

A total of 162 cancer patients referred for various cancer therapies (chemo/surgery/combination therapies) were included. All patients were administered the HADS. The association between anxiety scores and various factors such as age, site, and sex was found using the chi-square test.

Results

Thirty-nine (24.1%) patients had severe anxiety, 60 (37%) patients had borderline anxiety, and 63 (38.9%) patients were found to be normal. Fifty-three (32.7%) patients had severe depression, 47 (29%) patients had borderline depression, and 62 (38.3%) patients were found to be normal. The findings of this study indicate that educational status and occupational status are the significant factors (p < 0.05) responsible for increasing the risk of prevalence of anxiety and depression in cancer patients. Another interesting observation in this study was that patients with breast and gastrointestinal cancer had the highest prevalence of depression among other cancer types.

Conclusions

The present study contributed to the prevalence of anxiety and depression in cancer patients in Tamil Nadu, India. While the study population is small, which is a limitation of the present study, it has provided an overview that educational status and occupation contribute significantly to anxiety and depression, which has not been explored much in the past. To efficiently manage this, patients should be made aware of the financial support available from various philanthropic groups, government policies, and insurance so that they can improve their quality of life and manage their clinical condition in a more confident manner. These findings call for the need for early psychiatric interventions in cancer care to improve the quality of life of patients by focusing on improving patients' mental stability and adherence to the medications for providing positive outcomes from the cancer treatments.

## Introduction

Cancer is a severe and life-threatening disease. Because of the multiple risks associated with the disease, including the diagnosis of a potentially lethal illness, complicated treatment regimens, and the resulting side effects, cancer is typically a stressful experience for patients [1,2]. People who have been diagnosed with cancer frequently experience depression and anxiety [3,4]. Untreated psychiatric comorbidities in cancer patients can significantly increase morbidity, resulting in poor medication adherence, longer and also more recurrent hospitalizations, a short life expectancy, a lower standard of living, and higher mortality [5]. The majority of the data emerged from developed countries, where socioeconomic and demographic circumstances differ from those in developing and underdeveloped countries.

The life of a cancer patient may be significantly impacted by anxiety and depression, but literature on emotional distress in the Indian cancer community is scarce [4]. In addition, the influence of psychosocial variables in this population, including age, gender, financial status, and emotional distress, remains uncertain.

We performed a search using the keywords “anxiety and depression in cancer patients AND Indian population,” which resulted in 17 results when the search was restricted to the past 10 years. Out of these, only four studies [6-9] were exclusively conducted on Indian patients with various cancer types wherein anxiety and depression were evaluated.

With this background and the scarcity of studies on psychological parameters, including anxiety and depression in the Indian population, more specifically in the state of Tamil Nadu, clinically diagnosed with cancer, the present study was initiated.

This cross-sectional, quantitative observational study was aimed at finding the prevalence of anxiety and depression in cancer patients attending a tertiary care center in Tamil Nadu, India. The levels of anxiety and depression in these patients were correlated with various factors, such as age, sex, marital status, educational status, occupation, financial support, duration, type of care, sort of carcinoma, and stages of malignancy.

## Materials and methods

Study design and location

This cross-sectional, quantitative observational study was aimed at finding the prevalence of anxiety and depression in cancer patients attending the G. Kuppuswamy Naidu Memorial Hospital, Coimbatore, Tamil Nadu, India from July 2022 to December 2022, using the Hospital Anxiety and Depression Scale (HADS).

Inclusion criteria

Patients above 18 years of age, whether male or female, with clinically diagnosed cancer of any type, were included in this study.

Exclusion criteria

Patients with a history of psychiatric diseases well before the clinical diagnosis of cancer were excluded from the study.

Ethical clearance and informed consent

The study was approved by the Institutional Ethics Committee of G. Kuppuswamy Naidu Memorial Hospital (Approval #: IEC/GKNMH/4M/19) and informed consent was obtained from the participants. All the authors confirm that no animal subjects or tissue were used in this work.

Assessment instrument

Hospital Anxiety and Depression Scale (Approved by the WHO)

All patients were administered the HADS, which is a 14-item scale grouped into two subscales, i.e., anxiety and depression, with seven items each (depression for even items and anxiety for odd ones). The items are measured using a Likert scale ranging from 0 to 3, with higher scores indicating more severe anxiety and depression. The guidelines established by Zigmond and Snaith [10] were used to calculate the scores. A score of 0 to 7 indicates no symptoms, a score of 8 to 10 indicates a borderline case, and a score of 11 to 21 indicates clear symptoms of depression and/or anxiety.

Data were collected via a face-to-face interview employing a structured and pre-tested form specifically designed for the study. Two sections were enclosed within the questionnaire: (A) individual characteristics and socio-demographic factors (age, place of residence, legal status, and academic level); (B) clinical data (sort of therapy, psychotropic medicine used, treatment regimens, stage of cancer, current activity, and symptoms burden).

Data analyses

Data were statistically analyzed using SPSS (IBM Corp., Armonk, NY). The Pearson chi-square test was used to measure the relationship between HADS individual demographic parameters and clinical variables. The p-value was set at 0.05.

## Results

A total of 162 patients were identified to be eligible for participation in this study and they were administered the HADS questionnaire.

Table 1 represents the summary of the socio-demographic status of the study population. The average age (± SD) of the participants was 54.82 ± 13.54 years (range: 19-90 years). Most patients were in the age group of 51-60 years. Of the participants, 101 (62.3%) were female and 61 (37.7%) were male. A small proportion of the study’s participants (6, 3.7%) were unmarried, and 156 (97.3%) were married. In terms of educational level, 19.1%, 23.45%, 35.18%, and 22.2% were illiterate, primary school, senior high school, and graduates, respectively. The majority of patients (72.8%) paid their own feasible care costs, while insurance covered 27.1% of therapy management expenses.

**Table 1:** Sociodemographic status of the patients (n = 162) * Chi-square test p-value < 0.05 statistically significant. HADS: Hospital Anxiety and Depression Scale.

Study variables	Category	Frequency	Percentage	The p-value of the HADS score	
				Anxiety	Depression
Age in years	Less than 20	02	1.2	0.484	0.291
	21-30	09	5.6		
	31-40	07	4.3		
	41-50	34	21.0		
	51-60	50	30.9		
	61-70	42	25.9		
	71-80	13	8.0		
	81-90	05	3.08		
Gender	Male	61	37.7	0.475	0.989
	Female	101	62.3		
Marital status	Married	156	96.3	0.834	0.183
	Unmarried	101	3.7		
Educational status	Uneducated	31	19.1	0.060	0.046*
	Primary school	38	23.4		
	High school	57	35.1		
	University	36	22.2		
Occupational status	Non-working	54	33.3	0.006*	0.010*
	Working	108	66.6		
Financial support	Insurance	44	27.1	0.650	0.187
	Own	118	72.8		

More than half (33.33%) of the participants participating in the study had a disease period of less than six months, followed by 22.83% of participants with a disease duration of less than one to two years. Most of the patients (60.5%) were scheduled for chemotherapy and the others for surgery or combination therapies. The most involved carcinoma site was that of the breast (33.9%), followed by gastrointestinal (17.3%), gynecological (8.6%), and lung carcinoma (9.2%). The study enrolled 53 (32.71%) stage I, 63 (38.88%) stage II, and 46 (28.39%) stage III cancer patients out of 162 patients based on the cancer stage (Table 2).

**Table 2:** Basic characteristics of cancer patients HADS: Hospital Anxiety and Depression Scale.

Study variables	Category	Frequency	Percentage	The p-value of the HADS score	
				Anxiety	Depression
Duration	˂6 months	54	33.3	0.285	0.839
	6 months to 1 year	12	7.40		
	1-2 year	37	22.8		
	2-3 year	29	17.9		
	˃3 years	30	18.5		
Type of care	Palliative care	42	25.9	0.178	0.348
	Surgery	19	11.7		
	Radiation	03	1.85		
	Chemotherapy	98	60.5		
Sort of carcinoma	Lymphoma	10	6.17	0.203	0.06
	Sarcoma	08	4.93		
	Leukemia	10	6.2		
	Lung	15	9.2		
	Breast	55	33.9		
	Gastrointestinal tract	28	17.3		
	Gynecological	14	8.64		
	Mouth	07	4.3		
	Prostrate	05	3.1		
	Others	10	6.2	0.552	0.127
Stages of malignancy	I	53	32.7		
	II	63	38.9		
	III	46	28.4		

**Figure 1:**
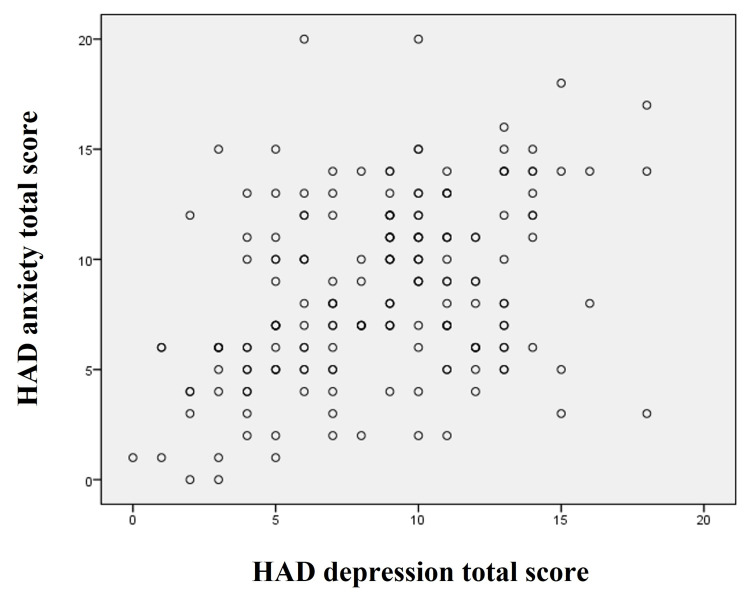
Correlation of the Hospital Anxiety and Depression (HAD) scale scores

The study analyzed using the HADS indicated that 76 (46.91%) of 162 patients suffered from anxiety disorder alone, 54 (33.33%) suffered from depression, and 32 (19.75%) suffered from both anxiety disorders and depression. Based on the HADS score in the anxiety category, patients had more borderline (37%) than severe anxiety (24%). In the case of depression, severe depression (32.7%) was higher than borderline depression (29%) among patients using the HADS depression category (Table 3). The results findings have shown a positive correlation observed with total anxiety and depression score (Table 4 and Figure 1).

**Table 3:** HADS scores of the study sample HADS: Hospital Anxiety and Depression Scale.

Category	No. of patients	Percentage
Anxiety score		
Normal (0-7)	63	38.9
Borderline (8-10)	60	37.0
Severe (11-21)	39	24.1
Depression score		
Normal (0-7)	62	38.3
Borderline (8-10)	47	29.0
Severe (11-21)	53	32.7

**Table 4:** Correlation of HADS scores ** Correlation is significant at the 0.01 level (two-tailed). HADS: Hospital Anxiety and Depression Scale.

HADS parameters	HADS anxiety total score	HADS depression total score
HADS anxiety total score, Pearson correlation	1	0.601^**^
Sig. (2-tailed)	-	0.000
n	162	162
HADS anxiety total score, Pearson correlation	0.601^**^	1
Sig. (2-tailed)	0.000	-
n	162	162

## Discussion

This cross-sectional, observational study was aimed at finding the prevalence of anxiety and depression in cancer patients and correlation of anxiety and depression with various factors such as age, sex, marital status, educational status, occupation, financial support, duration, type of care, sort of carcinoma, and stages of malignancy, among cancer patients attending the G. Kuppuswamy Naidu Memorial Hospital, Coimbatore, Tamil Nadu, India from July 2022 to December 2022, using the HADS.

A total of 162 cancer patients referred for various cancer therapies (chemo/surgery/combination therapies) were included. All patients were administered the HADS. The association between anxiety scores and various factors was statistically analyzed using the chi-square test. Out of the 162 patients, 76 suffered from anxiety, 54 suffered from depression, and 32 suffered from both anxiety and depression. Based on the HADS score, patients with borderline anxiety were higher than the severe category. However, patients with severe depression were more than those with borderline depression.

In the present study, patients with breast cancer and gastrointestinal cancer showed increased anxiety and depression. The fact that breast cancer patients are at high risk for developing psychiatric disorders such as depression and anxiety is consistent with the reported literature [11-15]. The prevalence of depression and anxiety was 42.6% and 40.9%, respectively, in a cross-sectional study involving 425 cancer patients from the Butaro Cancer Center of Excellence. Cancer patients who started chemotherapy had a higher chance of being depressed than those who started chemotherapy and counseling. Compared to Hodgkin's lymphoma, breast cancer was found to considerably increase the incidence of depression [16]. Other cancer types have also been reported to cause an increase in anxiety and depression. In a study with 100 Indian patients with various types of cancer, maximum anxiety and depression were observed in patients with head and neck cancer; in addition, older age was a major contributing factor leading to anxiety and depression [8].

In the present investigation, among the various factors studied, occupation and educational status were the significant (p < 0.05) factors contributing to anxiety and depression in the studied cancer population. These factors are directly related to the financial status of the affected individual and given the uncertainty of today’s world, post-COVID-19, it is affirmative to believe that occupation and educational status contribute to anxiety and depression in cancer patients. Some patients were taking antidepressants and antipsychotic drugs to manage their mental health and quality of life, while others did not.

Incorporating mental therapies into a cancer patient's treatment plan can help control psychiatric illnesses, particularly anxiety and despair, and is recommended as a national health policy by some authors [17-19]. The observations of the present study also reiterate that psychological therapies, including counseling, should begin right from the identification of the disease.

## Conclusions

The present study contributed to the prevalence of anxiety and depression in a population of cancer patients in Tamil Nadu, India. While the study population is small, which is a limitation of the present study, it has provided an overview that educational status and occupation contribute significantly to anxiety and depression, which has not been explored much in the past. To efficiently manage this, patients should be made aware of the financial support available from various philanthropic groups, government policies, and insurance so that they can improve their quality of life and manage their clinical condition in a more confident manner. These findings call for the need for early psychiatric interventions in cancer care to improve the quality of life of patients by focusing on improving patients' mental stability and adherence to the medications for providing positive outcomes from the cancer treatments.
